# The Role of Biomarkers in Surveillance of Ulcerative Colitis-Associated Colorectal Cancer: A Scoping Review

**DOI:** 10.3390/jcm14175979

**Published:** 2025-08-24

**Authors:** Justin Kritzinger, Gynter Kotrri, Peter L. Lakatos, Talat Bessissow, Gary Wild

**Affiliations:** 1 Department of Medicine, McGill University, Montreal, QC H3G 2M1, Canada; 2 Department of Medicine, Division of Gastroenterology, McGill University, Montreal, QC H3G 2M1, Canada; gynter.kotrri@mail.mcgill.ca (G.K.); peter.lakatos@mcgill.ca (P.L.L.); talat.bessissow@mcgill.ca (T.B.); gary.wild@mcgill.ca (G.W.); 3 Department of Internal Medicine and Oncology, Semmelweis University, 1083 Budapest, Hungary

**Keywords:** ulcerative colitis, colorectal cancer, tissue biomarkers, dysplasia surveillance, molecular risk stratification

## Abstract

Ulcerative colitis (UC) is associated with an elevated risk of colorectal cancer (CRC), driven by chronic inflammation and a distinct inflammation–dysplasia–carcinoma pathway. Conventional surveillance relies on colonoscopy and histologic assessment, but flat, multifocal dysplasia and sampling limitations challenge early detection. Tissue-based biomarkers offer promise in improving risk stratification and identifying patients at high risk for UC-associated CRC (UC-CRC). This review explores key categories of tissue biomarkers with potential clinical utility, including genetic mutations, epigenetic alterations, microRNA expression profiles, and markers of genomic instability such as telomere shortening, copy number variants, and aneuploidy. Many of these molecular alterations precede histologic dysplasia and reflect a “field effect,” suggesting their potential role in early cancer detection. Despite compelling associations between these biomarkers and neoplastic progression, most lack prospective validation and are not yet ready for routine clinical use. Future research should prioritize the development of integrated biomarker panels and validate their predictive accuracy in longitudinal UC cohorts. Molecular profiling may ultimately enable personalized, risk-adapted surveillance strategies that improve early detection while minimizing unnecessary interventions.

## 1. Introduction

Ulcerative colitis (UC) is an idiopathic, chronic, and progressive inflammatory disorder affecting the colon, characterized by continuous mucosal inflammation from the rectum to the proximal colon [1]. The risk of colorectal cancer (CRC) after 20 years of disease duration has been estimated as high as 5–10% and individuals with UC have a 2.48-fold higher risk for colorectal cancer in comparison to the general population [2,3,4,5,6,7]. The pathogenesis of colitis-associated CRC (UC-CRC) differs significantly from that of sporadic CRC, following an inflammation–dysplasia–carcinoma sequence rather than the classic adenoma–carcinoma progression [8,9]. While endoscopic surveillance with random and targeted biopsies remains the gold standard for dysplasia detection in UC, it has inherent limitations, including sampling error, low dysplasia yield, low compliance, interobserver variability in histopathological interpretation, and challenges in distinguishing dysplasia from inflammatory regenerative changes [10,11]. Moreover, surveillance is resource-intensive, and subtle lesions may evade detection due to their flat morphology or the presence of a field effect, wherein genetically altered but histologically normal-appearing mucosa gives rise to neoplasia [5]. These limitations underscore the need for more precise and sensitive approaches, such as molecular biomarkers, to complement traditional screening strategies.

Tissue-based biomarkers have shown significant potential in addressing these gaps. Genetic mutations, particularly in *TP53*, are frequently observed in early colitis-associated neoplasia and may even precede histological dysplasia [5,11]. Additionally, epigenetic changes, such as DNA methylation of tumor suppressor genes and mismatch repair gene promoters, are also early and widespread events, contributing to the field effect seen in patients with UC [5,6]. Aneuploidy, reflecting chromosomal instability, has also been correlated with progression to high-grade dysplasia and carcinoma, and is emerging as a possible prognostic indicator [10,12]. Lastly, several microRNAs (miRNAs) have been identified as early markers of neoplastic changes and hold promise for risk stratification [5,12].

This scoping review sought to examine the current landscape of tissue-based biomarkers for their utility in CRC screening among patients with UC. Firstly, we will discuss the pathogenesis of CRC in UC. Secondly, we mapped out the available evidence and identified gaps in point mutations, aberrant methylation changes, and microRNA dysregulation in UC-associated CRC. We aimed to inform future research directions and support the notion of integrating tissue-based biomarkers into personalized surveillance strategies for this high-risk population.

## 2. Materials and Methods

**Figure 1:**
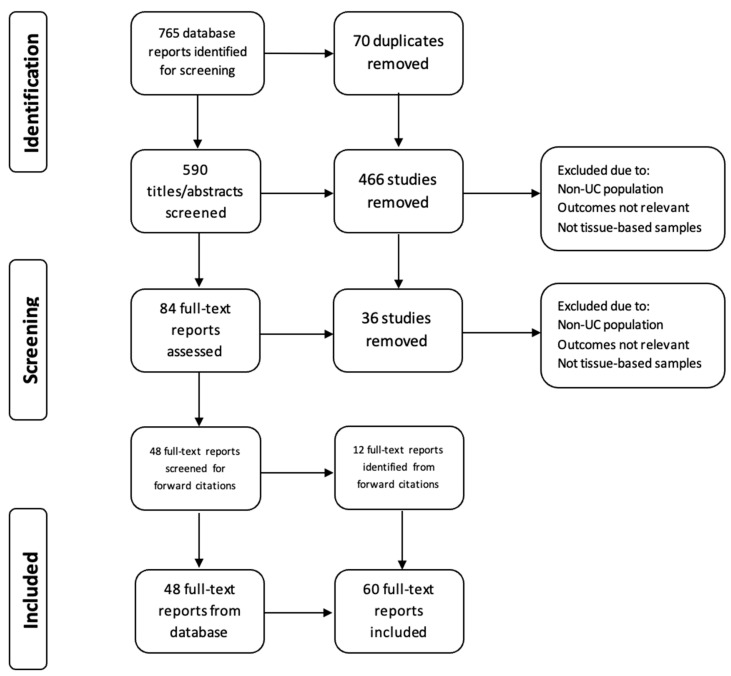
The PRISMA flow diagram details our search and selection process applied during the review. An initial search strategy revealed 765 reports, from which 48 full texts were identified following title, abstract, and text screening. A total of 60 reports were included after forward citations were screened.

**Figure 2:**
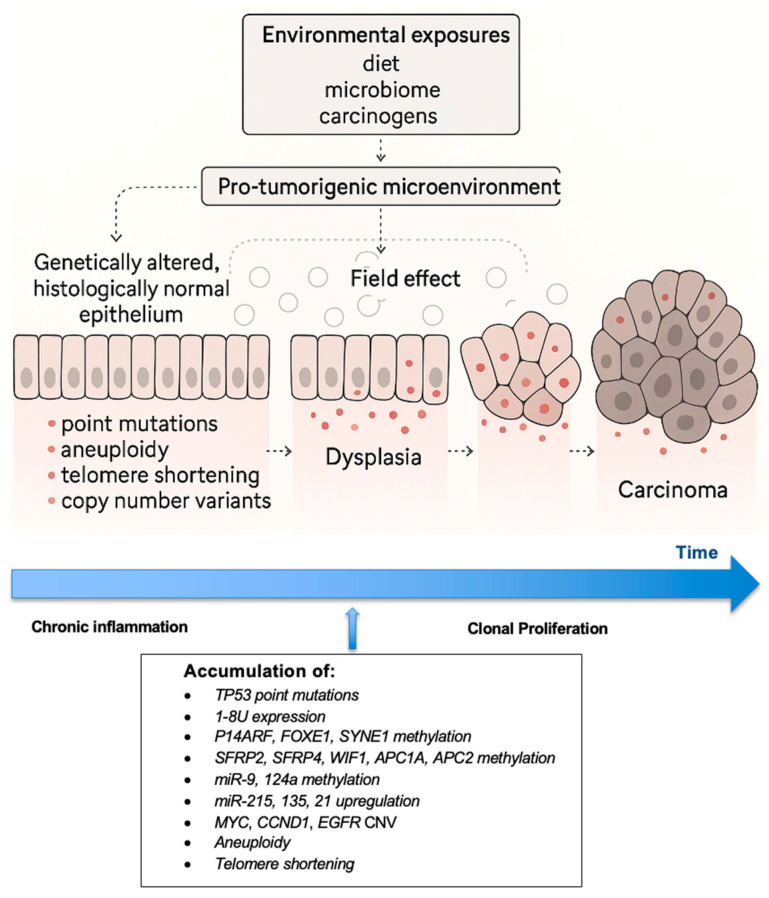
The “field effect” demonstrating how dysplastic and neoplastic tissue develops within histologically normal tissue through the accumulation of early *TP53* mutations, epigenetic alterations, and development of aneuploidy and telomere shortening, ultimately resulting in cancerous lesions.

The study design of a scoping review was considered most suitable to explore multiple, emerging key concepts for CRC risk in inflammatory bowel disease (IBD) [13]. A literature search was conducted on 31 March 2025 using the electronic database PubMed, OVID/Medline to identify relevant English language articles published between 1984 and 31 March 2025. In addition, reference lists of the identified articles were screened for additional studies. The Preferred Reporting Items for Systematic Reviews and Meta-analysis (PRISMA) extension for scoping reviews was followed. The PRISMA-ScR checklist is available on the Open Science Framework at https://osf.io/6trkq/files/osfstorage/6882be44292dd116a3242196 (accessed on 15 March 2025). The following search strategy was used: ((“Colitis, Ulcerative” [Mesh]) OR (Ulcerative colitis OR IBD OR UC OR Inflammatory Bowel Disease OR Colitis OR Proctocolitis OR Pancolitis)) AND (Tumor marker* OR Tumour marker* OR *TP53* OR *KRAS* OR oncogene* OR telomere* OR microRNA OR miRNA OR mutation OR biomarker*) AND (Colorectal cancer OR colon cancer OR rectal cancer OR polyps OR dysplasia OR neoplasia OR ulcerative colitis-associated neoplasia) AND (Field effect OR screening OR surveillance). This initial search identified 765 articles, of which 124 were identified following duplication removal and title screening, and 84 articles were identified following abstract screening. Original articles were considered eligible if (1) molecular biomarkers were assessed in patients with UC, (2) biomarkers were related to clinical outcomes, and/or (3) biomarkers were obtained via colorectal biopsy. Articles were excluded if they were non-human studies or did not examine biomarkers from colorectal tissue. Finally, 60 articles were included in this review (Figure 1). Data from included studies were charted using a standardized data extraction form developed a priori by the review team. The form was piloted on a subset of eligible articles to ensure consistency and comprehensiveness in capturing relevant study characteristics, biomarker types, detection methods, and clinical outcomes. Data charting was conducted independently by two reviewers, with discrepancies resolved through discussion or adjudication by a third reviewer. ChatGPT 4.0 was used in the partial image generation of Figure 2. Specifically in Figure 2, the image conveying the development of the field effect was generated via ChatGPT 4.0, and the authors completed the image denoting the timeline and accumulation of various mutations.

## 3. Pathogenesis

Colorectal cancer is a well-recognized complication of longstanding UC, arising via a distinct inflammation-driven pathway that differs from the sporadic adenoma–carcinoma sequence [8,9]. In UC, chronic relapsing inflammation initiates a cascade of molecular alterations that promote carcinogenesis across a broad field of the colonic mucosa—a phenomenon known as the “field effect” (Figure 1) [5]. The main predictors clinically are extent, severity of inflammation, duration, and probably age at onset of UC. Thus, this field of genetically altered histologically normal tissue provides fertile ground for multifocal neoplastic transformation.

The earliest pathogenic changes are linked to sustained oxidative stress. Inflammatory cells in UC release reactive oxygen and nitrogen species (ROS and RNS), causing direct DNA damage in epithelial cells [6,14]. Oxidative DNA injury contributes to point mutations, strand breaks, and chromosomal instability, ultimately promoting neoplastic transformation. Furthermore, aberrations in immune surveillance, such as disrupted epithelial cell–T-cell crosstalk via CD80, are implicated in the progression from low-grade dysplasia to cancer [6]. Importantly, these effects are compounded by the activation of pro-tumorigenic signaling pathways such as IL-6/STAT3 and TNFα/NF-κB, which enhance epithelial cell survival, proliferation, and resistance to apoptosis [15].

A defining early event in UC-associated carcinogenesis is the mutation of the tumor suppressor gene *TP53* [15]. Unlike in sporadic CRC, where *TP53* mutations typically occur later in the mutation cascade, in UC, they are frequently found in non-dysplastic or early dysplastic mucosa, suggesting a role in initiating neoplastic change [6,16]. Overexpression of the interferon-inducible gene *1-8U* has also been observed in severely inflamed and cancerous UC mucosa, indicating its potential utility in distinguishing high-risk areas before morphological dysplasia appears [16]. Conversely, mutations in *APC* and *KRAS*, which are early events in sporadic CRC, are less common or delayed in UC-CRC, emphasizing the divergence in molecular pathogenesis [15,17].

Epigenetic dysregulation also plays a pivotal role. Promoter hypermethylation of tumor suppressor genes and mismatch repair genes leads to gene silencing and contributes to both microsatellite instability and chromosomal instability [6,14]. It has been proposed that the increased oxidative stress in patients with UC results in enough DNA damage to exceed the capacity of repair mechanisms, eventually leading to the accumulation of DNA damage and microsatellite instability. Additional molecular changes in keeping with accelerated aging, such as telomere shortening and aneuploidy, further destabilize the genome, promoting progression from low-grade to high-grade dysplasia and ultimately to invasive carcinoma [18].

Baker et al. have described three conceptual types of the field effects including (1) etiologic (environmental exposures, diet, microbiome, and genetic factors promoting a microenvironment of cancer susceptibility), (2) molecular (point mutations, aneuploidy, telomere shortening, etc), and (3) morphologic (normal tissue, dysplasia, cancer) [5]. Clinically, dysplasia in UC is often flat or invisible, making detection challenging. These lesions tend to arise diffusely within areas of active or previously inflamed mucosa rather than from discrete polyps [4,19]. Notably, flat dysplastic lesions exhibit higher levels of genomic instability, including aneuploidy and widespread DNA copy number alterations, compared with visible polypoid lesions or sporadic adenomas [19]. Furthermore, patients with early onset UC (defined as UC arising between ages 25 and 35) who develop CRC have been shown to have extensive fields of molecular abnormalities throughout their colons compared with patients who have late onset of disease (defined as UC arising between ages 55 and 65) [5]. Specifically, large clonal populations with shortened telomeres have been identified within multiple non-dysplastic areas of the colon among patients with UC with high-grade dysplasia or cancer, yet almost never in those who had late onset of disease [5]. This diffuse and unpredictable pattern reinforces the importance of a field effect as a central concept in UC carcinogenesis [5].

The pathogenesis of UC-CRC is multifactorial, driven by multiple factors including chronic inflammation, oxidative DNA damage, early *TP53* mutations, epigenetic silencing, and genomic instability, as summarized in Figure 2. The field effect and the presence of flat, genomically unstable dysplasia further distinguish UC-CRC from its sporadic counterpart, underscoring the need for enhanced molecular surveillance strategies.

## 4. Point Mutations

Genetic mutations, particularly in tumor suppressor genes and oncogenes, are central to the pathogenesis of UC-CRC [20,21]. Among the most studied genetic mutations in UC-CRC are those affecting the *TP53* and *KRAS* genes. These mutations not only delineate the molecular evolution of UC-CRC but also offer opportunities for early detection and risk stratification through tissue-based surveillance.

*TP53* mutations are widely acknowledged as early events in the neoplastic progression of UC, and numerous studies have shown that *TP53* mutations are frequently detected in histologically normal or inflamed mucosa, preceding dysplasia and carcinoma [22,23,24,25,26,27,28,29,30,31,32]. Using next-generation sequencing (NGS), Singhi et al. examined genetic mutations associated with dysplastic and neoplastic tissue in IBD. Alterations in *TP53* were detected in 71% of specimens with low-grade dysplasia, 83% of those with high-grade dysplasia, and 100% in colorectal adenocarcinoma. By comparison, no mutations of TP53 or other genes were identified within uninvolved colonic tissue [29]. Brentnall et al. further demonstrated that mutations at codon 248 of *TP53* were found not only in dysplastic and neoplastic tissues but also in adjacent non-dysplastic mucosa, indicating clonal expansion and the phenomenon of a field effect in UC-CRC development [25]. This was corroborated by Hirsch et al., who found that *TP53* mutations were present in 87% of UC-CRC cases, a higher frequency than observed in sporadic CRC (61%). These mutations were often unique across different tumor sites in the same patient, suggesting multiple independent neoplastic events driven by chronic inflammation [27].

Further, studies employing immunohistochemistry (IHC) for *TP53* have demonstrated that its overexpression correlates strongly with dysplasia and cancer. For example, Xie et al. observed a progressive increase in *TP53* nuclear staining from negative mucosa through low- and high-grade dysplasia to carcinoma. They found that combining *TP53* overexpression with cytokeratin 7 positivity significantly improved diagnostic specificity for dysplasia in UC patients [28]. Horvath et al. expanded on these findings by demonstrating that p53 overexpression in mucosa indefinite for dysplasia predicted subsequent progression to neoplasia in 25% of patients, reinforcing its role as an early biomarker of malignant potential [30].

Fuji et al. further demonstrated the potential of IHC analysis to detect *TP53* alterations [33]. In their study, 59.5% (25 of 42) of the neoplastic lesions (dysplasia and carcinoma) and 40.0% of the lesions that were indefinite for dysplasia displayed nuclear accumulation of p53 protein. Thus, IHC analysis of p53 could serve as a useful marker of neoplasia, particularly where discrimination between neoplasia and regenerative epithelium is difficult [33]. However, the authors importantly note that not all mutations (e.g., nonsense or frameshift mutations) result in the accumulation of the p53 protein in the nucleus. In fact, approximately 93% of neoplastic lesions that displayed negative IHC staining for p53 protein demonstrated a *TP53* mutation within exons 5–8 under PCR single-stranded conformation polymorphism, suggesting increased sensitivity of PCR methods for detecting UC-CRC [33]. In future clinical practice, PCR detection of *TP53* mutations should be considered for pathologic identification of UC-CRC, rather than incorporating IHC methods.

*KRAS* mutations, in contrast, appear less frequently and tend to occur later in the sequence of UC-CRC progression. In a meta-analysis by Du et al., *KRAS* mutations were significantly less common in UC-CRC compared with sporadic CRC (RR = 0.71), whereas *TP53* mutations were more frequent (RR = 1.24) [20]. Studies analyzing colonic lavage fluid have found *KRAS* mutations in a minority of UC patients, often co-occurring with *TP53* mutations and typically in those with longer disease duration [34]. Given that *KRAS* mutations have not been shown to be a significant predictor of dysplasia and have low specificity in stool and tissue samples, they are less likely to play a role in future clinical practice [34,35].

The integration of these mutations into colorectal cancer surveillance strategies offers promise. *TP53* mutations, detectable through PCR-based methods, IHC, or even in colonic lavage fluid, serve as early indicators of neoplastic transformation [22,23,24,25,26,27,28,29,30,31,32]. Importantly, detection in non-dysplastic mucosa underscores their utility in identifying high-risk patients even before histologic changes occur. This is particularly valuable in UC, where dysplasia may be multifocal, flat, and easily missed during routine colonoscopy.

## 5. Methylation Patterns

Aberrant DNA methylation plays a critical role in the pathogenesis of UC-CRC, functioning as a key epigenetic mechanism that contributes to the silencing of tumor suppressor genes during the inflammation–dysplasia–carcinoma sequence [33,36,37,38]. Methylation changes, particularly promoter hypermethylation of specific genes, are detectable in non-neoplastic mucosa and are often associated with long-standing disease and extensive colitis, suggesting their potential as early biomarkers of neoplastic transformation [36,38].

One of the earliest and most well-studied epigenetic alterations involves the *p14ARF* gene. In a prospective study, hypermethylation of *p14ARF* was found in 100% of dysplastic tissues and 26% of rectal biopsies from patients without histologic dysplasia, indicating its presence as an early, potentially pre-dysplastic event. Importantly, individuals with *p14ARF* hypermethylation were significantly more likely to develop dysplasia during surveillance compared with those without such methylation, underscoring its predictive value for future neoplastic progression [36].

Similarly, hypermethylation of the tumor-suppressive miRNA gene miR-124a has been identified as a potential biomarker for use in CRC risk stratification among patients with UC. In one study, elevated methylation levels of miR-124a-3 were correlated with known risk factors, such as pancolitis and long disease duration. Patients with both pancolitis and long-standing UC had 7.4-fold higher methylation levels than those without these risk factors [37]. Similarly, miR-9 methylation has been shown to be significantly higher in neoplastic tissue compared with normal tissues [39]. These findings highlight the potential of epigenetic markers such as DNA methylation that may serve as a quantitative measure of cumulative carcinogenic risk and can potentially distinguish between patients at low and high risk for UC-CRC.

Hypermethylation of *SYNE1* and *FOXE1*, two genes involved in cell cycle regulation and tumor suppression, has been investigated as a possible biomarker for risk stratification for the development of UC-CRC [38]. In a retrospective study by Papadia et al., these genes showed significantly increased promoter methylation in colorectal tissue from patients with dysplasia and UC-CRC compared with those with non-dysplastic colitis and healthy controls [38]. Whereas hypermethylation of both *FOXE1* and *SYNE1* was absent among controls, promoter hypermethylation was detected in biopsies of 60% of patients with UC-CRC for *FOXE1* and 80% for *SYNE1*. Hypermethylation was increasingly likely with increased disease severity, indicating that it may be a specific marker of malignant transformation in the setting of chronic inflammation [38]. Increased methylation of additional genes such as *ER*, *BMP3*, and *NDRG4* has been identified as a possible marker of high risk for the development of UC-CRC [33]. Interestingly, high levels of *ER* gene methylation have been found not only in regions with neoplasia but also in other areas widely dispersed throughout the colorectum. These results suggest that a single biopsy sample may suffice when attempting to identify high-risk individuals, minimizing the requirement of numerous biopsy samples [33].

The ENDCAP-C study sought to validate previously identified biomarkers of neoplasia in a retrospective cohort and create predictive models for later validation in a prospective cohort [40]. In a study including 35 patients with cancer, 78 with dysplasia, and 343 without neoplasia undergoing surveillance for UC-CRC across six medical centers, a multiplex methylation panel including five markers (*SFRP2*, *SFRP4*, *WIF1*, *APC1A*, *APC2*) was accurate in detecting pre-cancerous and invasive neoplasia (AUC = 0.83; 95% CI: 0.79, 0.88), and dysplasia (AUC = 0.88; (0.84, 0.91). In the setting of non-neoplastic mucosa, modest accuracy was achieved (AUC = 0.68; 95% CI: 0.62, 0.73) in predicting associated bowel neoplasia through the methylation patterns of distant non-neoplastic colonic mucosa [40]. This limitation suggests that, although these panels capture some early molecular alterations, their ability to reliably identify high-risk patients without visible or histologic changes is constrained, underscoring the need for refinement with additional biomarkers to improve predictive accuracy in UC-CRC surveillance.

Methylation markers may address current limitations in endoscopic surveillance, such as sampling error and interobserver variability in interpreting indefinite or low-grade dysplasia. Epigenetic testing could help stratify patients more precisely, allowing personalized surveillance intervals based on molecular risk rather than histologic findings alone. DNA methylation patterns—particularly those involving genes like *p14ARF*, miR-124a, miR-9, *SYNE1*, and *FOXE1*—represent a promising avenue for enhancing CRC surveillance in ulcerative colitis.

## 6. microRNA

MicroRNAs (miRNAs) have emerged as pivotal molecular regulators in the pathogenesis of UC-CRC. These small non-coding RNAs function post-transcriptionally by binding to target mRNAs to suppress gene expression [41,42]. miRNAs play an important regulatory role in gene expression and protein translation and have been shown to impact the expression of oncogenes and tumor suppressor genes. In UC-CRC, chronic inflammation contributes to dysregulated miRNA expression, affecting key processes like cell proliferation, apoptosis, and immune response modulation [41]. This unique interaction between inflammatory signaling and miRNA dysregulation plays a fundamental role in the neoplastic transformation of the colonic epithelium. Thus, miRNA expression profiles in dysplastic and neoplastic tissues remain a key area for further investigation and research, as elucidating specific miRNA profiles may serve to identify patients at high risk of developing UC-CRC [42].

Several miRNAs have been identified as potential tissue-based biomarkers of early neoplastic changes in UC. For instance, miR-21, an oncogenic miRNA, is significantly upregulated in inflamed mucosa and even more elevated in UC-CRC, where it likely promotes inflammation-associated carcinogenesis by enhancing proliferative and anti-apoptotic pathways [43]. miR-135b also shows a stepwise increase in expression from non-dysplastic to dysplastic and then to neoplastic tissues in UC, positioning it as a possible biomarker for tracking malignant progression [43].

Profiling studies have demonstrated distinct miRNA expression signatures across different stages of neoplastic progression in UC. For example, differential expression of miR-192, miR-194, and miR-215 has been observed between UC and UC-CRC tissues, highlighting their utility in distinguishing neoplastic from inflamed but non-cancerous tissue [44,45]. In particular, miR-215 has been shown to be significantly upregulated in non-dysplastic mucosa 1 to 5 years prior to the onset of neoplasia in patients with long-standing UC (defined as UC for greater than 10 years) [44]. This implies a field effect where molecular changes precede histologic abnormalities. Its elevated expression in UC-CRC and adjacent non-dysplastic mucosa compared with normal controls underlines its role in early tumorigenesis and its potential as a predictive biomarker for cancer development [44]. miR-9, while often studied in the context of methylation, is notable for its epigenetic silencing in UC-CRC. Studies have shown that miR-9 methylation increases with age, disease duration, and proximity to cancer, and is significantly higher in rectal mucosa from UC-CRC patients compared with controls [39]. Its methylation status can distinguish cancer from non-neoplastic tissues with high accuracy (AUC: 0.94), suggesting that its epigenetic downregulation may be both a marker and a mechanistic contributor to carcinogenesis [39].

The clinical implications of these findings are substantial. Given their stability in formalin-fixed tissues and even biofluids, miRNAs are ideal candidates for minimally invasive surveillance tools [46]. They offer the potential to complement or even surpass current histologic approaches, which are limited by sampling error and interpretive variability. Incorporating miRNA signatures into surveillance protocols could enable earlier detection of at-risk mucosa, guide colonoscopy intervals, and tailor preventive interventions to those at highest risk.

## 7. Other Biomarkers

Additional biomarkers of interest, such as telomere shortening, copy number variation (CNV), and aneuploidy, offer valuable insights into the molecular pathogenesis of UC-CRC and serve as promising tools for cancer surveillance in this high-risk population. These markers reflect genomic instability, a key feature of the inflammation-dysplasia-carcinoma sequence unique to UC-CRC.

Telomere shortening, often driven by chronic inflammation, plays a significant role in early carcinogenic processes. Salk et al. demonstrated that patients with early-onset UC who developed neoplasia exhibited significantly shorter telomeres in non-dysplastic mucosa compared with individuals who did not develop neoplasia [47]. These patients also had an increased prevalence of clonal expansions—clonal mutations in polyguanine tracts resulting in fields of genetically altered epithelium—suggesting that telomere erosion may precede and facilitate clonal proliferation and malignant transformation. In this study, clonal expansions were associated with proximity to dysplasia, and the mean percentage of clonally expanded mutations distinguished early-onset progressors from non-progressors with 100% sensitivity and 80% specificity. These results convey the potential of clonal expansions as potential biomarkers for surveillance, particularly in younger patients with extensive disease [47].

Copy number variations are a hallmark of chromosomal instability and are increasingly recognized in UC-associated neoplasia. A study by Shivakumar et al. using array comparative genomic hybridization identified distinct CNVs in UC-associated neoplasia compared with sporadic CRC. Genes such as *MYC*, *CCND1*, and *EGFR* were amplified in dysplastic and neoplastic tissues, and their expression levels correlated with disease progression [48]. These CNVs, especially when validated through qRT-PCR and immunohistochemistry, provided moderate predictive value for detecting neoplasia in UC, and may ultimately complement histopathologic evaluation [48].

Aneuploidy, or abnormal DNA content, has long been associated with malignancy and is a strong indicator of genomic instability in UC [49]. Meling et al. analyzed DNA ploidy in dysplastic and non-dysplastic mucosa of patients with UC, and found that DNA aneuploidy was present not only in carcinomas but also in flat mucosa and dysplastic tissues—often in areas devoid of histologic abnormalities [49]. Importantly, aneuploidy was observed in patients who subsequently developed metastatic CRC, underscoring its prognostic relevance. When comparing histologic dysplasia to DNA flow cytometry, cytometric analysis offers the advantage of higher intra-observer reliability in interpreting DNA histograms. Rubin et al. proposed that aneuploidy detected in biopsies classified as indefinite for dysplasia may forecast future progression [50]. In their prospective study of 25 high-risk patients with UC, five of six individuals with aneuploidy developed dysplasia within 1–2.5 years, whereas none of the nineteen patients without aneuploidy progressed during the same period. Similar findings were reported by Lofberg et al., who observed that aneuploidy preceded, coincided with, or followed the development of dysplasia in a cohort of 59 patients [50]. Importantly, mutations were not confined to dysplastic lesions but were detected throughout the colon in patients who eventually developed dysplasia. In some cases, mutations were identified during endoscopic procedures in areas without histologic evidence of dysplasia. This could reflect either sampling error or widespread genomic instability. The latter is supported by other molecular markers of instability, including p16 promoter methylation, elevated telomerase activity, and chromosomal aberrations detected by comparative genomic hybridization in resected colons from patients with UC-CRC [50]. Given its occurrence in non-dysplastic tissue, DNA aneuploidy may serve as an adjunctive biomarker, identifying patients who might benefit from intensified surveillance despite the absence of histologic dysplasia [49,50,51].

The integration of telomere shortening, CNVs, and aneuploidy into UC surveillance strategies offers a molecularly informed approach that can overcome the limitations of conventional histology. These biomarkers not only precede visible dysplasia but also reflect underlying genomic alterations, enabling early detection of cancer risk and personalized surveillance protocols for patients with long-standing UC.

## 8. Discussion

Tumorigenesis in UC is recognized as a multistep process, progressing from low-grade dysplasia to high-grade dysplasia and ultimately carcinoma [47,49]. As discussed in this review, several studies have described the occurrence of a field effect, whereby neoplastic lesions arise from colonic tissue displaying molecular or genetic alterations that precede dysplastic changes [5,18,52]. Current ACG surveillance guidelines recommend that individuals with UC and a disease duration exceeding eight years undergo evaluation with colonoscopy every one to two years for the early detection of dysplasia, while ECCO and BSG utilize stratified guidelines that determine the frequency of surveillance [53,54,55,56]. Despite advances in endoscopic imaging, such as the use of chromoendoscopy, studies have not shown a direct effect in preventing all-cause/cancer-specific mortality or time to interval cancer [57]. However, the efficacy of this approach is limited by the morphologic characteristics of UC-associated dysplasia, which is frequently flat and endoscopically inconspicuous [19]. As a result, surveillance typically relies on systematic 4-quadrant random biopsies taken at 10 cm intervals throughout the colon. While this strategy is intended to enhance histologic detection, it is inherently resource-intensive, invasive, and reliant on probabilistic sampling. For example, a previous (2005) survey of British gastroenterologists found that only 24% conducted surveillance colonoscopy in patients with left-sided colitis, while only 2% routinely took more than 20 biopsies and only 53% recommended colectomy when high-grade dysplasia was identified [58]. Consequently, it may preferentially detect more extensive or advanced lesions while missing focal or early neoplastic changes. While sampling error from spatial heterogeneity limits the detection of focal molecular changes, the utilization of the field effect may mitigate these limitations. These widespread biochemical changes precede visible lesions and increase the likelihood of detection in random biopsies, enhancing the utility of molecular biomarkers. By capturing early, diffuse alterations, biomarker-based surveillance may overcome sampling constraints inherent to conventional histology in UC-CRC; however, a certain degree of sampling error will likely remain. Incorporating such biomarkers into routine screening could improve early risk stratification, reduce procedural burden, and enhance the precision of colorectal cancer surveillance in UC.

Numerous biomarkers have been proposed as potential candidates to further improve colorectal cancer screening in patients with UC, as discussed in this review and summarized in Table 1. While this review does not represent an exhaustive overview of all biomarkers proposed (see Chen et al. for a comprehensive overview), promising markers are discussed, and the need for additional prospective research is highlighted [7]. For example, *TP53* mutations represent a robust early biomarker of neoplastic progression in UC-CRC. Detection in non-dysplastic tissue supports their role in a field effect, and use as an adjunctive biomarker to identify patients at high risk of developing UC-CRC who may benefit from additional screening. Additional genetic markers, including aneuploidy, telomere shortening, CNVs, epigenetic methylation, and miRNA expression patterns, hold promise in their ability to stratify patients more precisely. Their association with disease duration, extent, and progression to dysplasia underscores their value not only as early markers of carcinogenesis but also as a potential to inform personalized surveillance intervals based on molecular risk rather than histologic findings alone.

Recent evidence supports the clinical utility of genomic biomarkers in risk stratification for UC-associated neoplasia. In a multicenter case-control study, Al Bakir et al. demonstrated that low-pass whole genome sequencing (lpWGS) of low-grade dysplasia (LGD) lesions in patients with UC can robustly predict progression to advanced neoplasia (high-grade dysplasia or colorectal cancer) [59]. They studied 270 LGD samples and found that the burden of somatic copy number alterations (CNAs) was significantly greater in patients who progressed. A genomic CNA score, derived from features such as chromosome 17q loss and microsatellite instability, showed superior predictive performance compared with conventional clinicopathologic factors. When combined with clinical variables like incomplete resection, the multivariate model achieved an AUC of 0.95 at 5 years. These findings suggest that CNA profiling via lpWGS could serve as a scalable and cost-effective adjunct to current surveillance protocols, enabling more precise identification of high-risk patients and reducing unnecessary colectomies [59].

The emerging utility of liquid biopsies as biomarkers offers a minimally invasive and dynamic surveillance alternative to tissue-based methods in detecting UC-CRC. As comprehensively reviewed by Ma et al. (2024), liquid biopsy components—including circulating tumor cells (CTCs), circulating tumor DNA (ctDNA), exosomes, and tumor-educated platelets—are readily detectable in blood or other body fluids, enabling real-time monitoring of tumor progression, molecular heterogeneity, and early malignant transformation [60]. These modalities are particularly appealing for UC-CRC surveillance, where frequent colonoscopic biopsies are burdensome. Notably, ctDNA can reveal genetic and epigenetic alterations reflecting evolving dysplasia or neoplastic change, while CTCs and exosomes may capture viable tumor characteristics or secreted molecular signatures [60]. Looking ahead, artificial intelligence promises to enhance surveillance by integrating multi-dimensional liquid biopsy data—such as mutational profiles, exosomal RNAs, or platelet-derived signals—into predictive algorithms. Machine learning could improve early detection, risk stratification, and individualized monitoring protocols, offering a complementary, non-invasive layer of biomarker-driven surveillance in UC-CRC.

**Figure 3:**
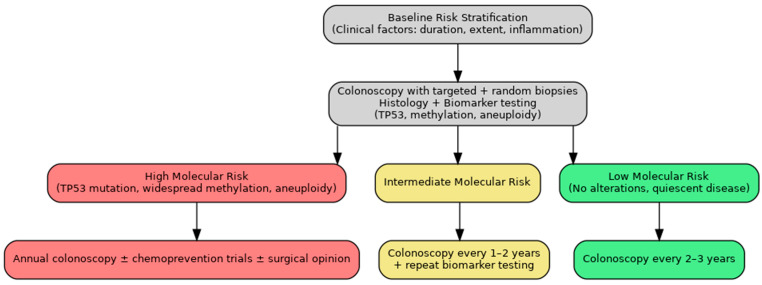
Proposed biomarker-integrated surveillance algorithm for UC-CRC. Baseline risk stratification is followed by colonoscopy with histologic and biomarker analysis (panel of biomarkers including *TP53* mutations, high-risk methylation patterns, aneuploidy, and other identified markers). High-risk profiles prompt annual surveillance ± chemoprevention and surgical consultation for consideration of colectomy depending on overall identified risk. Intermediate-risk individuals will repeat colonoscopy with biomarker testing every 1–2 years, and low-risk individuals every 2–3 years. This approach aims to improve early detection while reducing unnecessary procedures in low-risk patients.

While biomarker-based surveillance for UC-CRC holds promise, cost-effectiveness, and feasibility data are limited. Assays such as PCR-based *TP53* mutation detection, methylation panels, and low-pass whole genome sequencing are increasingly accessible; however, their integration into routine surveillance requires formal economic evaluation and standardization. Implementation would necessitate validated cut-offs, reproducible assays, and integration with existing endoscopic workflows. As described in Figure 3, we propose a theoretical algorithm for the implementation of such screening practices.

This review offers a structured synthesis of the current literature on tissue-based biomarkers for colorectal cancer surveillance in ulcerative colitis. The strengths of this review include employing a scoping review methodology aligned with PRISMA-ScR guidelines, allowing us to systematically capture a wide range of emerging molecular targets—including genetic, epigenetic, and transcriptomic biomarkers—across nearly four decades of research. The inclusion of diverse biomarker categories such as *TP53* mutations, DNA methylation patterns, microRNA profiles, telomere shortening, and aneuploidy reflects the multifactorial nature of UC-associated carcinogenesis and highlights the evolving landscape of precision surveillance. By emphasizing clinically relevant biomarkers with direct applicability to endoscopic practice, it bridges the gap between molecular research and real-world surveillance strategies. The inclusion of emerging concepts such as the field effect and risk-adapted algorithms further strengthens its translational value for personalized patient care.

The scoping review process employed in this study carries several limitations. First, while scoping reviews are well-suited for mapping broad research areas and identifying knowledge gaps, they do not typically include formal assessment of study quality or risk of bias, limiting the ability to draw definitive conclusions about the strength of evidence. Additionally, the literature search was confined to English-language studies, potentially introducing language bias and omitting relevant findings from non-English sources. The inclusion of studies spanning over four decades (1984 to 2025) may also introduce heterogeneity due to evolving diagnostic techniques, molecular assays, and surveillance practices. Lastly, as with all narrative syntheses, there is a risk of subjective interpretation during data selection and thematic categorization, which may affect reproducibility and generalizability.

## 9. Conclusions

Numerous tissue biomarkers with the potential to identify high-risk patients for ulcerative colitis-associated colorectal cancer (UC-CRC) have been discussed in this review. Given their association with dysplasia and widespread presence across colonic mucosa, molecular alterations hold significant promise for improving risk stratification beyond current histologic methods. However, at present, the integration of biomarkers into routine surveillance is limited by a lack of high-quality, prospective evidence confirming their predictive accuracy. Histologic evaluation remains the cornerstone for dysplasia detection.

Future research must prioritize the development and validation of biomarker-based risk models in large, longitudinal UC cohorts. These models should aim to identify patients at greatest risk of neoplastic progression and inform surveillance intensity accordingly. We recommend that future studies investigate composite panels incorporating genetic mutations (e.g., TP53), epigenetic changes (e.g., methylation patterns), miRNA signatures, copy number variants, and aneuploidy. Integration with clinical and endoscopic data may enhance predictive performance and facilitate a precision medicine approach to surveillance.

Ultimately, risk-adapted surveillance protocols that incorporate validated biomarkers could reduce unnecessary colonoscopies in low-risk individuals while enabling earlier detection and intervention in those at highest risk. This approach has the potential to improve patient outcomes and optimize resource utilization in inflammatory bowel disease care.

## Figures and Tables

**Table 1:** Summary of key tissue-based biomarkers identified in UC-CRC and their clinical relevance as possible markers in UC-CRC screening.

Analyte	Biomarker	Clinical Relevance	Reference
DNA	*TP53*	*TP53* mutations are prevalent in UC-associated dysplasia and cancer, detected in 71–100% of lesions, but absent in uninvolved mucosa. These mutations often occur in non-dysplastic tissue, supporting a field effect. *TP53* overexpression on IHC correlates with dysplasia and neoplasia and improves diagnostic specificity when combined with CK7. While some mutations evade IHC detection, PCR analysis detects *TP53* mutations in 93% of IHC-negative neoplastic lesions, indicating its value as a sensitive molecular marker.	[5,15,16,20,21,22,23,24,25,26,27,28,29,30,31,32,33]
	*KRAS*	*KRAS* mutations are less frequent in UC-CRC than sporadic CRC, occur later in tumor progression, and often co-occur with *TP53* mutations. Due to their low predictive value for dysplasia and poor specificity in stool and tissue samples, *KRAS* mutations likely have limited clinical utility in UC-CRC surveillance.	[20,34,35]
	Aneuploidy	In a prospective study of 25 high-risk UC patients, five of six individuals with aneuploidy developed dysplasia within 1–2.5 years, whereas none of the nineteen patients without aneuploidy progressed during the same period. Similar findings were reported by Lofberg et al., who observed that aneuploidy preceded, coincided with, or followed the development of dysplasia in a cohort of 59 patients [51].	[10,12,18,48,49,50,51]
	*1-8U*	IFN-inducible gene *1-8U* was highly expressed in UC-associated cancers and chronically inflamed UC mucosa but absent in normal tissue. Its expression was independent of disease duration or extent.	[16]
	Telomere shortening	Shortened telomeres and clonal expansions were common in non-dysplastic mucosa of early-onset UC Progressors, distinguishing them from non-progressors with high sensitivity and specificity. These changes, absent in late-onset cases, suggest telomere shortening may serve as a biomarker for cancer risk in early-onset UC-associated colorectal cancer.	[5,47]
	Clonal expansions	Clonal expansions have been associated with proximity to dysplasia, and in one study, the mean percentage of clonally expanded mutations distinguished early-onset progressors from non-progressors with 100% sensitivity and 80% specificity.	[47]
	Copy number variations	Expression levels of amplified genes such as *MYC*, *CCND1*, and *EGFR* amplified in dysplastic and neoplastic tissues are correlated with disease progression. Low-pass whole genome sequencing (lpWGS) of low-grade dysplasia (LGD) lesions in UC patients can robustly predict progression to advanced neoplasia. A multivariate model achieved an AUC of 0.95 at 5 years.	[48,59]
microRNA	miR-21 miR-135b	miR-21 is significantly upregulated in inflamed UC mucosa and even more elevated in UC-CRC, where it likely promotes inflammation-associated carcinogenesis by enhancing proliferative and anti-apoptotic pathways. miR-135b shows a stepwise increase in expression from non-dysplastic to dysplastic and finally to neoplastic tissues in UC, positioning it as a possible biomarker for tracking malignant progression.	[43]
	miR-192 miR-194 miR-215	Differential expression of miR-192, miR-194, and miR-215 has been observed between UC and UC-CRC tissues, highlighting their utility in distinguishing neoplastic from inflamed but non-cancerous tissue. In particular, miR-215 has been shown to be significantly upregulated in non-dysplastic mucosa 1 to 5 years prior to the onset of neoplasia in patients with long-standing UC.	[43,44]
Methylation	*p14ARF*	In a prospective study, hypermethylation of *p14ARF* was present in all dysplastic tissues and 26% of non-dysplastic biopsies, suggesting it as an early, pre-dysplastic event. Its presence significantly predicted future dysplasia, supporting its potential role as a biomarker for neoplastic progression in ulcerative colitis.	[36]
	miR-124a	Elevated methylation levels of miR-124a-3 were correlated with known risk factors such as pancolitis and long disease duration. Patients with both pancolitis and long-standing UC had 7.4-fold higher methylation levels than those without these risk factors.	[37]
	miR-9	miR-9 methylation increases with age, disease duration, and proximity to cancer, and is significantly higher in rectal mucosa from UC-CRC patients compared to controls. Its methylation status has been used to distinguish cancer from non-neoplastic tissues with high accuracy (AUC: 0.94).	[39]
	*SFRP2*, *SFRP4*, *WIF1*, *APC1A*, *APC2*	Accurate in detecting pre-cancerous and invasive neoplasia (AUC = 0.83) and dysplasia (AUC = 0.88). For non-neoplastic mucosa, a four-marker panel (*APC1A*, *SFRP4*, *SFRP5*, *SOX7*) had modest accuracy (AUC = 0.68; 95% CI: 0.62, 0.73) in predicting associated bowel neoplasia through the methylation signature of distant non-neoplastic colonic mucosa.	[40]
	*SYNE1 FOXE1* *ER* *BMP3* *NDRG4*	Hypermethylation of *SYNE1* and *FOXE1* was detected in 80% and 60% of UC-CRC cases, respectively, but was absent in controls, correlating with disease severity. Additional hypermethylated genes (*ER*, *BMP3*, *NDRG4*) have also been linked to high UC-CRC risk. Single biopsy sampling may suffice due to widespread methylation patterns.	[33,38]

## Abbreviations

The following abbreviations are used in this manuscript:

UC	Ulcerative colitis
UC-CRC	Ulcerative colitis-associated colorectal cancer
IBD	Inflammatory bowel disease
CRC	Colorectal cancer
ROS	Reactive oxygen species
RNS	Reactive nitrogen species
NGS	Next-generation sequencing
IHC	Immunohistochemistry
CNV	Copy number variation
lpWGS	Low-pass whole genome sequencing
